# Editorial: “We Know What We Are, but Know Not What We May Be”: Metacognition and Self-Awareness in the Aging Brain

**DOI:** 10.3389/fpsyt.2021.745071

**Published:** 2021-08-31

**Authors:** Maria Donata Orfei, Josep Garre-Olmo, Sergio Starkstein

**Affiliations:** ^1^Molecular Mind Laboratory (MoMiLab), IMT School for Advanced Studies Lucca, Lucca, Italy; ^2^Girona Biomedical Research Institute (IDIBGi), Girona, Spain; ^3^School of Psychiatry and Clinical Neurosciences University of Western Australia, Crawley, WA, Australia

**Keywords:** aging, self-awarness, anosognosia, metacognition, cognitive decline and dementia

The worldwide increase in life expectancy compels a progressively larger population segment to face new demands as the integration of ongoing physical, social, cognitive, and emotional changes into the previous self-knowledge can be highly challenging for the elderly. Metacognition, or the knowledge and reflective capacity one has concerning one's cognitive functioning, is an essential skill in the elderly. It underpins self-awareness and facilitates successful adaptation. However, the detrimental effects of altered metacognition, particularly of anosognosia, on quality of life in the elderly and the clinical outcome of cognitive decline forms, such as Alzheimer's Disease (AD), have earned increasing attention. The goal of this Research Topic was to update state-of-the-art and enrich our knowledge of metacognitive processes in the elderly in a multifaceted frame, based on original neuropsychological, neuroanatomical, genetic, and neuropsychiatric evidence. In this Special Issue, the contributions are mainly focused on, but not limited to, anosognosia in dementia.

Two studies face the understanding of the predictive role of self-awareness defects in the early stages of dementia. The longitudinal study by Bastin et al. investigated the degree of awareness of cognitive difficulties in Mild Cognitive Impairment and adopted a multidimensional approach. Patients enrolled in the research underwent questionnaires and FDG-PET, and structural MRI. Anosognosia could be an early sign of neurodegeneration in brain areas that would support control mechanisms over memory representations. On the other hand, Bellaali et al. combined genetic data, in particular Apolipoprotein E (APOE ε4), self-appraisal, and spouses' rating of the level of the participant's memory functioning. They highlighted that subjective memory complaints were less predictive than spouse-appraised memory functioning of a subsequent memory decline. Both of these studies cast light on the vulnerability of metacognition even in non-demented elderly subjects as a prelude to cognitive decline.

Another crucial aspect investigated was the relationship between anosognosia and neuropsychiatric symptoms. In the last decade, several studies showed significant evidence of the co-occurrence of impaired awareness and specifically depressive, anxiety, and apathy symptoms in AD, but some inconsistencies persist in the literature. In this issue, Azocar et al. performed a systematic review of high-quality studies on this topic and in particular in mild to moderate AD. Their results support previous evidence on the strength of the association between apathy and anosognosia, while the role of depressive and anxiety symptoms is downsized. Tondelli et al. provide another point of view challenging the same question but considering the age at onset of illness. Thus, they investigated the association between anosognosia and neuropsychiatric symptoms in different forms of dementia and, particularly, compared the course of disease in early-onset vs. late-onset patients. Although the level of anosognosia was not significantly different between EOD and LOD, it increased over time with disease progression and was higher in patients affected by pathological processes of the frontal cortical areas. Moreover, the relationship between apathy and anosognosia is further supported, especially in early-onset dementia.

Finally, two papers challenge self-awareness as a process related to aging, introducing new original theoretical models to interpret these complex phenomena. Hughes and Touron focus on constructing and developing subjective age as a self-awareness product, not necessarily corresponding to the lived years. Moreover, the authors illustrate the strict relationship between contextual influences and subjective age and how it affects behaviors and well-being in different situations and moments of life. The model is fascinating for its multidimensional bio-psycho-social frame.

Bomilcar et al., on the other hand, provide an ambitious model aimed at best defining the otherwise often evanescent concept of the self. It encompasses seven fundamental dimensions or processes of the self: an embodied self, an agentic self, an implicit self, a critical self, a surrogate self, an extended self, and an emergent self, each catching a specific function of self-awareness and metacognitive processes, finally merging in a unitary concept and subjective experience of the self. Far from being a purely theoretical proposal, the authors discuss their seven-self model as underpinned by neuroscience and social psychology evidence and suggest it may contribute to understanding pathological phenomena such as anosognosia in dementia.

We want to thank all the authors who joined this initiative to have made their solid and long-lasting expertise available in this work.

We are confident that this special issue will represent an advanced step toward defining a multifaceted and comprehensive study of metacognition in the elderly and, in particular, of anosognosia in cognitive decline. Moreover, we believe that all the contributions included offer several stimuli and pave the way to further fruitful studies in the field.

## Author Contributions

MDO conceived and wrote the work; JGO and SS revised and approved it for the publication and agreed to be accountable for all aspects of the work.

## Conflict of Interest

The authors declare that the research was conducted in the absence of any commercial or financial relationships that could be construed as a potential conflict of interest.

## Publisher's Note

All claims expressed in this article are solely those of the authors and do not necessarily represent those of their affiliated organizations, or those of the publisher, the editors and the reviewers. Any product that may be evaluated in this article, or claim that may be made by its manufacturer, is not guaranteed or endorsed by the publisher.

